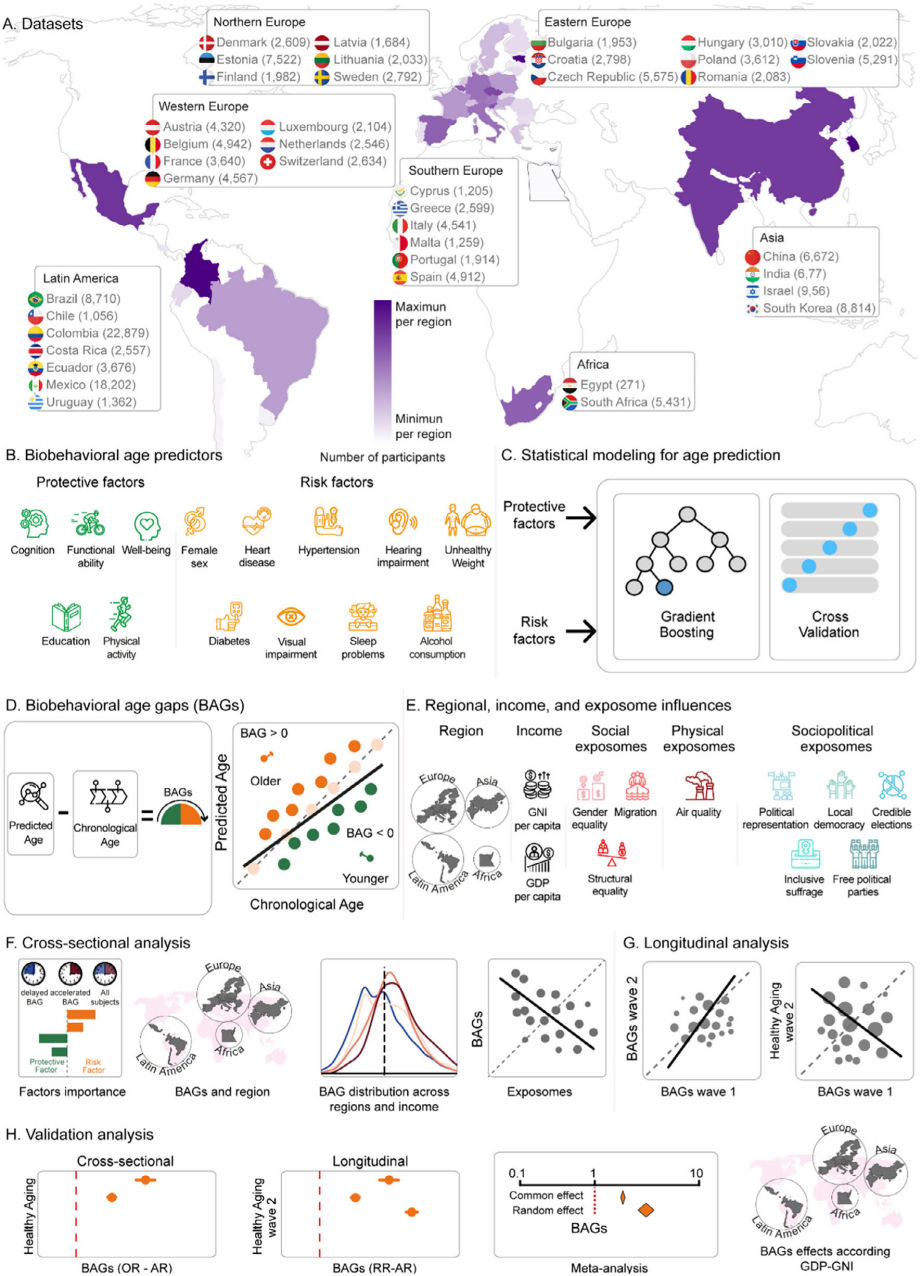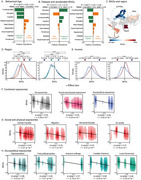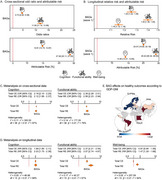# Biobehavioral Age Gaps as a Global Index to Assess Disparities and the Exposome in Accelerated Aging Across 40 Countries

**DOI:** 10.1002/alz70860_096545

**Published:** 2025-12-23

**Authors:** Agustin Ibanez

**Affiliations:** ^1^ Latin American Brain Health Institute (BrainLat), Universidad Adolfo Ibañez, Santiago, Chile

## Abstract

**Background:**

An interplay of protective and risk factors influences healthy aging, especially in high‐inequality regions. The cumulative effects of these factors on delayed and accelerated aging remain underexplored, especially regarding socioeconomic and multimodal exposome disparities. This study introduces biobehavioral age gaps (BAGs), a novel framework to measure the combined effect of protective and risk factors and their modulation by disparities and exposomes. By incorporating protective and risk factors and global modulation of disparities and exposome, BAGs may offer ecological and cost‐effective solutions applicable to public policy and capacity‐building efforts in underrepresented regions.

**Methods:**

BAGs were estimated with machine learning from 161,981 participants (45·09% females, mean age=67·06, SD=9·85) across 40 countries. National surveys from Latin America (LA), Europe, Asia, and Africa incorporated protective (preserved cognition, functional ability, education) and risk factors (cardiometabolic conditions, female sex, sensory impairments) for healthy aging and dementia. Cross‐sectional and longitudinal analyses assessed BAGs by regions, incomes, and physical (air quality), social (gender inequality, socioeconomic inequality), and sociopolitical (political representation, local democracy) exposomes. Validation analyses included odds ratios, attributable risk, relative risk, and metanalyses.

**Results:**

BAGs predicted age, with protective factors linked to delayed (*R* ^2^=0·57, r=0·75, RMSE=6·51) and risk factors to accelerated aging (*R* ^2^=0·69, r=0·84, RMSE=5·44). Africa and LA exhibited more accelerated aging than Europe and Asia. Low‐income countries showed more accelerated aging than high‐income nations (δd=0·20‐0·73; all *p* <0·0001). Within Europe, accelerated aging was higher in Eastern than Western regions and in Southern than Northern and Western regions (all δd=0.15–0.52, *p* <0.0001). Adverse physical, social, and sociopolitical exposomes were linked to accelerated aging (all d>0·37, *p* <0·0001). Higher baseline BAGs predicted future declines in functional ability (*r* = ‐0·33, *p* <1e‐15, d=0·70) and cognition (*r* = ‐0·22, *p* <1e‐15, d=0·44), as well as future BAGs (*p* <0·0001, d=1·55), indicating long‐lasting effects. Validation analysis confirmed these results.

**Conclusion:**

BAGs based in protective/risk factors capture accelerated and delayed aging and its modulation by socioeconomic and exposome disparities. BAGs provide actionable insights for public policy and capacity‐building initiatives by identifying modifiable factors to mitigate global aging disparities. Their ease of implementation supports targeted interventions and informed policymaking, especially in resource‐limited settings.